# Effects of application of rice husk biochar and limestone on cadmium accumulation in wheat under glasshouse and field conditions

**DOI:** 10.1038/s41598-022-25927-3

**Published:** 2022-12-19

**Authors:** Zehui Niu, Jiayan Ma, Xianzhi Fang, Zhaokun Xue, Zhengqian Ye

**Affiliations:** 1 State Key Laboratory of Subtropical Silviculture, Zhejiang A&F University, Hangzhou, 311300 Zhejiang China; 2 Key Laboratory of Soil Contamination Bioremediation of Zhejiang Province, Zhejiang A&F University, Hangzhou, 311300 Zhejiang China

**Keywords:** Biogeochemistry, Environmental chemistry, Environmental impact

## Abstract

Cadmium (Cd) has seriously threatened the safe production of food crops. Passivator amendments are commonly used to control the soil Cd availability. Yet, few studies are tested to explore the effect of the combination of various amendments. Here, we investigated the effects of different amendments (2% rice husk biochar, 2% limestone, and 1% rice husk biochar + 1% limestone) on the growth and Cd accumulation of wheat in pot and field experiments. The results showed that under the low soil Cd condition, the maximum increase of soil pH (1.83) was found in the limestone treatment compared to CK in pot experiment. Compared with the CK, the treatment of rice husk biochar decreased soil Cd availability and grain Cd content by about 25% and 31.2%, respectively. In contrast, under high soil Cd condition, the highest soil pH was observed in limestone, while the lowest soil Cd availability and grain Cd concentrations were found in rice husk biochar treatment. In the field experiment, the treatment of 1% rice husk biochar + 1% limestone caused a significant increase of soil pH by about 28.2%, whereas the treatment of 2% rice husk biochar reduced soil Cd availability and grain Cd content by about 38.9% and 38.5% compared to the CK. Therefore, rice husk biochar showed great potential to reduce Cd availability and ensure safe food production.

## Introduction

Cadmium (Cd) is one of the most toxic trace elements to the ecosystem and human beings, Cd contamination of soils is often difficult to be directly assessed or visually perceived, making it a hidden danger to the safe production of food crops. In 2014, the release of the Report on the national general survey of soil contamination by China officials raised alarm bells for all of us. The report showed that 16.1% of the farm land was over the standard for heavy metal soil contamination, and 7% of which was polluted by Cd in China. Soil heavy metal and especially Cd pollution also appeared in many other countries^1^. This excess Cd in farmland soil can easily migrate to crops and threaten human health via the consumption of agricultural products.

Wheat is one of the most important food crops in the world and wheat grains polluted with Cd has become an important concern issue worldwide^1^. Most previous reports indicated that wheat can easily uptake Cd from the soil. Through Zaid^2^ collation, the local area produces at least 150,000 kg of wheat that Cd levels 1.7–12.8-fold that far exceeds the national food standards every year. Xing et al.^3^ showed that grain concentrations of all 25 varieties for Cd exceeded the standard for consumption. While the wheat evolved tolerance mechanisms to alleviate the harmful effect of Cd stress, it was not enough for food safety^4^. There needs more measures to remediate contaminated soils to ensure the safe production of wheat grains.

Different strategies have been employed for remediating Cd- contaminated soil to decrease Cd uptake by crops, such as chemical, physical, and biological remediation techniques^5^. Chemical methods have been identified as a potential stock for remediation of Cd- polluted soils compared to other techniques due to their low cost of remediation and little impact on crops growth. The benefits of the chemical methods through application of organic and inorganic amendments have been widely demonstrated in remediating soils^6^. Among these amendments, limestone is cheap and effective to improve soil acidity, and effectively immobilizing Cd in soils^7^ and thus to reduce Cd uptake in plants^5^. For example, Zhou et al.^5^ found that the application of limestone significantly decreased Cd accumulation of wheat grain as a result of increase soil pH and decrease Cd availability. Besides, with the concept of ‘Carbon Sequestration’ proposed, biochar application has inevitably been a hot topic in Cd-polluted soil due to its achieving the carbon sequestration, utilizing the agricultural wastes, and ensuring the safety of consumption of agricultural products as well. In particular, rice husk biochar, as an organic amendment, is mainly applied to promote growth of crops^8–10^ and to remove metals from contaminated soil as a result of its characterized by high sources (FAO), higher content of silicon, and higher ash content^11^. According to the FAO online database, the total rice production was about 623 million tons, of which rice husk was about 125 million tons in Asia in 2008. Haefele et al.^12^ reported that the application of rice husk biochar increased wheat yield. The application of rice husk biochar at the rate of 3–12 t/ha resulted in soil pH increased usually in 1–2 units (Oladele et al.^13^), and decreased Cd-accumulation in wheat (Zheng et al.^14^). A combination of biochar and limestone was found to be more efficient in immobilizing heavy metal availabilities in the soil. Wang et al.^15^ showed that the highest reduction in soil DTPA—extractable Cd in the first year was observed with the application of combined biochar and limestone. In a pot experiment, Rehman et al. found that the greatest reduction of Cd in wheat grains was observed in limestone + biochar treatment^16^. Several other studies also showed that the combined application of amendments was more effective to mitigate soil Cd pollution in crops such as rice^16^ and wheat^16,17^. Numerous studies have reported that inappropriate application amendments might have the negative effect on soil properties. Long term application of limestone will lead to hardening^18^, and the increase of mineral nutrients leaching loss in the soil^19,20^. And some biochar was less effective for soil pH, so weakly alkaline biochar may be best for application to alkaline soils compare to acidic^21,22^.

Overall, few field studies are aimed at evaluating the effect of the combined application of amendments on decreasing metal uptake in wheat^23,24^, especially applying the biochar and limestone^25^. Although several studies have been carried out to evaluate the Cd-uptake in wheat with pot experiments^26,27^, and very little is studied in the field where there are more uncontrollable environmental factors^25,28,29^. Thus, more field studies and combined application of amendments are needed for achieving the safe eating of agricultural products in Cd contaminated soil.

In conclusion, this study was based on rice husk biochar and limestone at a same rate application, pot and field experiments were conducted to examine the efficiency of rice husk biochar and limestone applied alone or in combination to decrease Cd availability in soil and its uptake in wheat grains, to explore the suitable amendment and to offer important data reference into the safe utilization of Cd-contaminated soil with combined ameliorants.

## Materials and methods

A pot experiment was performed to investigate the effects of different treatments on accumulation of Cd in wheat under different levels of Cd (low and high) polluted soils. And a field experiment was conducted to examine Cd accumulation in wheat in a Cd-contaminated soil.

### Soil and amendments

The soil of the pot experiment was collected from a farmland in Jiaxin, Zhejiang Province. The soil was air-dried and ground to pass through a 2 mm sieve. Two levels of Cd, 3CdSO_4_·8H_2_O (GR grade) was added to the air-dried soil at 0.5 mg/kg (low Cd) and 1 mg/kg (high Cd). The mixture was homogeneously mixed and maintained moist at field capacity with deionized water for 3 months. The total Cd and available Cd in the soil were 0.58 mg/kg, 0.15 mg/kg, and 1.35 mg/kg, 0.48 mg/kg, respectively. The field experiment was conducted in a Cd-contaminated soil on the farmland of Wenzhou city, Zhejiang Province, China. The basic properties of the soils are given in Table 1.

**Table 1:** Basic physical and chemical properties of the test soils and biochar.

Property	Pot expt soil	Field expt soil	Rice husk biochar	Limestone
pH	6.21	5.98	8.32	8.6
Organic matter g/kg	31.36	31.54	–	–
Available N (mg/kg)	299.12	158.36	17.45	–
Available P (mg/kg)	44.86	81.87	26.85	–
Available K (mg/kg)	226	348	12.01	–
Total Cd (mg/kg)	0.23	0.64	No detected	No detected
Available Cd (mg/kg)	0.06	0.17	No detected	No detected
Total C (%)	–	–	47.5	–
Total N (%)	–	–	0.57	–
Total H (%)	–	–	2.78	–
Total O (%)	–	–	13.2	–
Surface area (m^2^g^−1^)	–	–	29.1	–
Ash (%)	–	–	30	–

The rice husk biochar and limestone used in the experiments were purchased from Jusanjiao Production Company, China. The rice husk biochar was made from rice husk powder with pyrolysis at 500 ℃ and then crushed through a 0.25 mm sieve.

The properties of rice husk biochar and limestone are listed in Table 1. The pH of rice husk biochar and limestone were determined by reference to the soil (1:20, w/v, weight to water ratio). Total hydrogen, carbon, and nitrogen contents were measured by using elemental analyzer (Elementar, Germany). The biochar alkalinity was determined with a back titration method.

### Pot experiment

A pot experiment was conducted in a greenhouse at Zhejiang A & F University. Sowing in December 2019 to harvest in May 2020, a total of six months. The experiment consisted of four treatments, namely, the control (no amendment addition, CK), 2% rice husk biochar (R), 2% limestone (L) and 1% rice husk biochar + 1% limestone (RL) with 3 replications of each treatment. Amendments were thoroughly mixed with 2.5 kg soil with 2.5 g of urea and 2.5 g of potassium dihydrogen phosphate per pot. The soil moisture was kept at about 70% of the field capacity using deionized water by weighing the pots every other day. Six wheat seedlings (Mianyou-1) were sown per pot and thinned to 3 seedlings^30^.

### Field experiment

The field experiment started in December 2019 in Wenzhou, Zhejiang Province, China and harvested in May 2020. Four treatments (control, 2% rice husk biochar, 2% limestone and 1% rice husk biochar + 1% limestone) were set up and each treatment had three replicates. Plot size was 3 m long × 1 m wide. Wheat grain seeds were sown in 15 columns with 20-cm line dispersing, 10–30 grains for every line. Daily management was the same as conventional field production.

### Sample collection and analysis

Plant and soil samples were collected at maturity. Plant samples were separated into roots, stems, sword leaves, other leaves, wheat husk and grains. Samples were washed with distilled water and then oven-dried at 70 °C till constant weight and dry weights were recorded before being milled for analysis. The soil was air dried and sieved by passing through a 2 mm sieve for analysis.

Soil pH was determined using a pH meter (FE20, Mettler Toledo, China). Alkaline hydrolysis nitrogen, available phosphorus, and available potassium were measured by the alkali-diffusion method, Olsen method, and ammonium acetate extraction-flame photometry method, respectively. Soil available Cd and total soil Cd content were determined by 0.1 mol/L HCl extraction and HF-HClO_4_-HNO_3_ digestion, respectively. Plant samples were digested with HNO_3_. Cd in the solutions was examined by ICP-OES (Optima 7000DV, Perkin Elmer Co. USA).

### Data statistics and analysis

All statistical analysis and mapping of data were performed using SPSS 26.0 and ORIGIN 2021.

## Result

### Pot experiment

#### Effect of rice husk biochar and/or limestone on soil pH and available Cd

As shown in the Table 2, compared to CK, all treatments increased soil pH while the degree of influence was different. The treatments of L and RL significantly increased the pH in soil by 34.6% and 32.1% compared to CK in low Cd soil. The results from the high Cd soil was similar to that in the low Cd soil, soil pH was significantly increased by19.3% and 20.4% under L and RL compared to CK. The availability of soil Cd significantly (*P* < 0.05) decreased by using amendments in both low Cd and high Cd soils compared to CK except for L in the low Cd soil. The greatest decrease in the availability of soil Cd was observed in R which was about 25% and 12.5% in low Cd soil and high Cd soil respectively. The availability of Cd in the soil was decreased by amendments in the following order R > RL > L > CK. Overall, the effect trend of the amendments was basically the same under different levels of soil Cd pollution.

**Table 2:** Effect of rice husk biochar and/or limestone on soil pH and available Cd.

Treatment		pH	Available Cd(mg/kg)
Low Cd	CK	5.30 ± 0.217c	0.16 ± 0.012c
	R	5.49 ± 0.137c	0.12 ± 0.020c
	L	7.12 ± 0.070a	0.15 ± 0.017c
	RL	7.00 ± 0.058a	0.14 ± 0.015c
High Cd	CK	6.08 ± 0.332b	0.48 ± 0.031a
	R	6.08 ± 0.100b	0.42 ± 0.035b
	L	7.31 ± 0.012a	0.47 ± 0.006a
	RL	7.25 ± 0.064a	0.46 ± 0.021ab

Data in the table are expressed as average ± standard deviation of three replicates.

Treatments include CK: no amendment addition, R: 2% rice husk biochar, L: 2% limestone, and RL: 1% rice husk biochar + 1% limestone.

Values followed with different letters indicate significantly different at *P* < 0.05.

#### Effect of rice husk biochar and/or limestone on wheat growth

In general, plant growth of wheat was inhibited by high Cd treatment compared to the low Cd, and it was affected by amendment treatments (Table 3). Except for L, the other treatments increased plant aboveground biomass and grain yield compared to the control. The highest increase in aboveground biomass and grain yield of wheat was obtained by R which was about 25.6% and 13.9%, 34.0% and 24.6% higher compared to CK respectively in the low Cd and high Cd soils. The root biomass of wheat was increased by amendment treatments in the following order R > RL > L > CK in low Cd soil. By contrast, in high Cd soil, the root biomass showed a decreasing trend by liming (L and RL treatments) and it was not affected by R. The application of rice husk biochar (R and RL treatments) showed a clear improvement on the aboveground biomass and grain yield of wheat regardless of soil Cd levels.

**Table 3:** Effect of rice husk biochar and/or limestone on biomass of wheat plants (g·plant^-1^).

Treatment		Biomass (g/plant)		
		Above ground	Root	Grain
Low Cd	CK	5.05 ± 0.13cd	0.21 ± 0.02d	1.94 ± 0.23bcd
	R	6.24 ± 0.01a	0.34 ± 0.01ab	2.60 ± 0.23a
	L	4.40 ± 0.21e	0.27 ± 0.01bcd	1.50 ± 0.40de
	RL	5.54 ± 0.17bc	0.29 ± 0.03bc	2.22 ± 0.12ab
High Cd	CK	4.37 ± 0.22e	0.37 ± 0.01a	1.74 ± 0.18cde
	R	5.75 ± 0.44ab	0.38 ± 0.05a	2.13 ± 0.20bc
	L	4.19 ± 0.52e	0.29 ± 0.05bc	1.44 ± 0.28e
	RL	4.85 ± 0.15de	0.23 ± 0.03 cd	1.73 ± 0.15cde

Data in the table are expressed as average ± standard deviation of three replicates.

Treatments include CK: no amendment addition, R: 2% rice husk biochar, L: 2% limestone, and RL: 1% rice husk biochar + 1% limestone.

Values followed with different letters indicate significantly different at *P* < 0.05.

#### Effect of rice husk biochar, limestone on Cd concentrations in wheat plants

**Figure 1:**
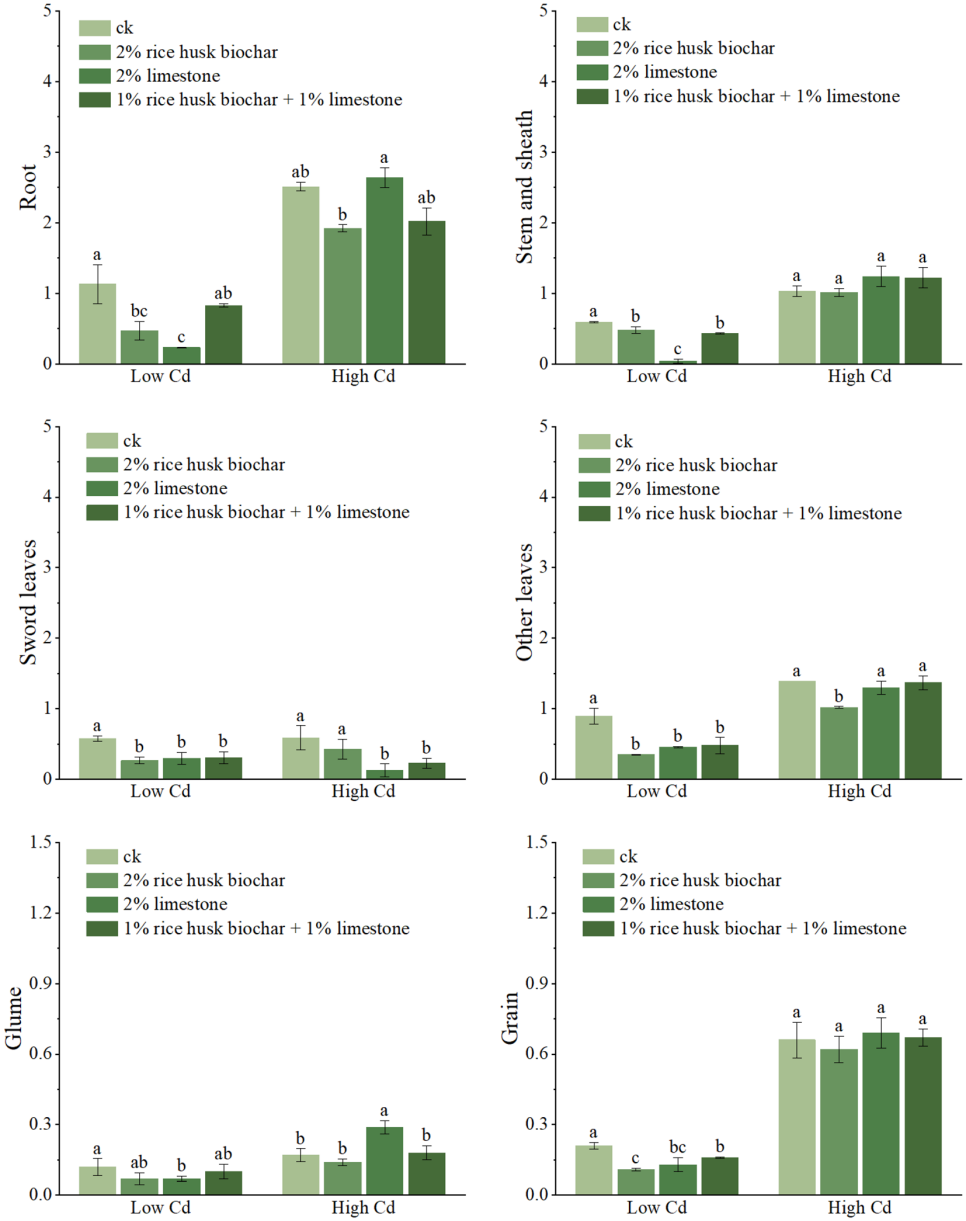
Effect of rice husk biochar and / or limestone on Cd content in different wheat organs (mg/kg). Values followed with different letters indicate significantly different at *P* < 0.05 determined by the Duncan. Treatments include CK: no amendment addition, R: 2% rice husk biochar, L: 2% limestone, and RL: 1% rice husk biochar + 1% limestone.

The concentrations of Cd in wheat under different treatments were observed as roots > other leaves > stems and sheaths > sword leaves > grains > glumes (Fig. 1). Cd concentrations in different plant parts were greatly reduced by amendment treatments in low Cd soil. The concentrations of Cd in roots, other leaves, stems and sheaths, sword leaves, grains, and glumes by R treatment were decreased by 58.4%, 61.7%, 19.7%, 53.8%, 47.6% and 40.4%, respectively, compared with control in the low Cd soil. Similarly, in the L and RL treatments, Cd concentrations were decrease by 79.6, 49.4, 41.0, 48.6, 38.1 and 4.5%; 26.5, 46.7, 28.1, 46.2, 23.85 and 21.1%. In the high Cd soil, the concentrations of Cd in roots, other leaves, sword leaves, grains, and glumes by R treatment were decreased by 31.5%, 32.5%, 28.2%, 5% and 17.6%, respectively, compared to the control. In high Cd soil, the Cd accumulation in wheat grains was 2.1–4.7 times higher than that in the low Cd soil, which far exceeded the standard of wheat safety production. In short, the R treatment showed a clear decrease of soil Cd availability and grain Cd content regardless of soil Cd level.

### Field experiment

Soil pH was significantly (*P* < 0.05) increased with the soil amendments compared to the control (Table 4). The greatest increase in pH was observed by RL which was about 1.39 units compared to the control. However, the R was the most effective in reducing available Cd contents in the soil which was about 38.9% compared to the CK. Similarly, the R also showed the greatest decrease of grain Cd content which was about 38.5% lower that the CK.

**Table 4:** Effect of rice husk biochar and / or limestone on soil pH and available Cd content, content of grain Cd.

Treatment		pH	Soil available Cd content(mg/kg)	Content of Cd in grain (mg/kg)
Field experiment	CK	5.03 ± 0.013c	0.18 ± 0.010a	0.26 ± 0.014a
	R	5.34 ± 0.003b	0.11 ± 0.003c	0.16 ± 0.008b
	L	6.42 ± 0.155a	0.13 ± 0.003b	0.23 ± 0.010a
	RL	6.45 ± 0.193a	0.14 ± 0.003b	0.22 ± 0.015a

Data in the figure are expressed as average ± standard deviation of the three replicates.

Treatments include CK: no amendment addition, R: 2% rice husk biochar, L: 2% limestone, and RL: 1% rice husk biochar + 1% limestone.

Values followed with different letters indicate significantly different at *P* < 0.05.

**Figure 2:**
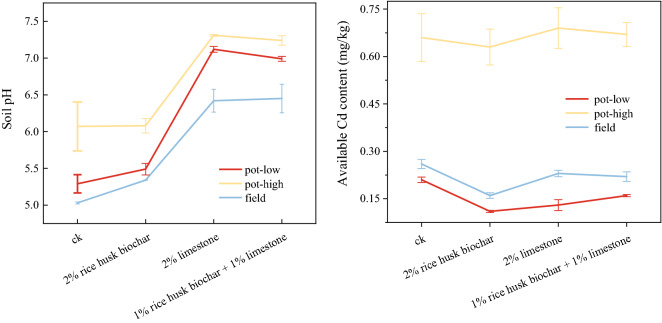
Dynamic changes of soil pH, available Cd concentration affected by different treatments. Treatments include CK: no amendment addition, R: 2% rice husk biochar, L: 2% limestone, and RL: 1% rice husk biochar + 1% limestone.

Observed the discovery of different trend lines (Fig. 2), under different experimental modes, soil pH followed the rules of L > RL > R > CK under amendments. But, in the field experiment, there was no significant difference between L and RL. Available Cd concentration in soil at two experiments under different treatments were similar, which showed in the order of R > RL > L > CK. Collation results suggested that R under any experimental condition could showed relatively high Cd-removing efficiency.

## Discussion

Single or combined application of amendments to the Cd polluted soil has been examined for a long time to see their effects on reducing plant Cd uptake. Numerous studies have reported that the uptake of Cd in the plant depends significantly on the bioavailability of Cd in the soil, while the bioavailability of Cd is controlled by soil pH, precipitation, absorption and complexation. In this study, it showed that all amendment treatments decreased the content of Cd in grains while the degree of influence was different. The lowest Cd content in grains was observed from rice husk biochar treatment, a decrease of 47.65% and 38.5% compared to CK in the low Cd soil and in the field. This was in consistent with its effect on soil Cd availability. The lowest soil availability of Cd was observed from rice husk biochar treatment, with 25%, 12.5%, and 38.9% decrease compared to CK in low Cd, high Cd, and field experiments. Many researchers have also reported similar results. In a study with Oak seedlings, Amirahmadi et al.^27^ reported that rice husk biochar amendments decreased the bioavailability of Cd, which was most significant at amendments rates of 3% and 5%. The decreased bioavailability of Cd in the soil was attibuted to complex mechanisms, such as precipitation with minerals, cation exchange, and complexation^23–26,31^. A high number of functional groups, pH, and porosity of rice husk biochar serve as an adsorbent of metals or precipitant. Bushra^28^ proposed that the biochar contains the aromatic functional group and hydroxyl group that interact with cation-Π and facilitate adsorption of metal cations^29,32^. Rehman et al.^33^ found that the lowest wheat grain Cd concentration was achieved by the application of rice husk biochar (96.5% compared to CK) at a rate of 2%. They also reported the increase in growth and biomass production after the application of biochar and the effect was attributed to the improvement of plant nutritional conditions and metal-binding characters of the biochar^34–36^. Furthermore, silicon has been shown to alleviate heavy metal stress mainly through formation of metal-silicate complexes via adsorption^4,37–40^. Thus, the silicon in rice husk biochar might also play an important role^4,41^. Notably, in our study, the highest increase in soil pH was obtained by limestone which was about 34.6%, 19.3%, and 27.6% compared to the control under the low Cd, high Cd, and field experiments. Numerous studies on limestone showed a significant enhancing effect on soil pH^42–44^. Liming materials could provide Ca^2+^ or Mg^2+^ cations to soil to neutralize soil acidity ^45^. Although the greatly improved soil pH by application of limestone, the effect on the content of Cd in wheat grains was irregular. This phenomenon could be due to many uncontrolled factors which were able to limit the outcomes of limes in soil^45–47^. That limestone reduced soil Cd activity could be largely due to its effect on raising soil pH. In contrast, besides increasing soil pH, the abundant function (ex: cation exchange, complexation, the porosity of biochar and silicate^40^) of rice husk biochar may be one of the reasons for the significant reduction in the bioavailability of Cd compared to limestone. Furthermore, Ramtahal et al.^48^ pointed out that the application of lime could induce zinc (Zn) deficiency for increased Cd uptake under high pH soils. In a previous study by Fageria^49^, the extractable Zn was decreased with increasing lime rate and the availability of Zn decreased 100-fold with each unit increase in pH. Moreover, Zn is an indispensable essential metal and micronutrient directly promoting the plant growth^50^. In a study by Adrees et al.^51^, with the increasing zinc oxide nanoparticles levels applied, soil bioavailable Cd content decreased while the wheat yield increased. In our experiments, although liming (L and RL treatments) resulted in great increase of soil pH and even higher than pH7.0 in the pot experiment, there was no significant difference in the available Cd in the soil compared to CK. Moreover, application of 2% limestone caused a significant decrease of wheat biomass. We deduce that the reason for the insignificant effect of limestone treatment on reducing available Cd in the soil and less effective on reducing Cd in the grains may partly be due to soil zinc deficiency induced by the excess of limestone. The effect of 1% rice husk biochar + 1% limestone was similar to 2% limestone but less effective than 2% rice husk biochar. Contrary, in rice experiments, soil pH change was similar to our experiments, rice grain Cd content was most decreased by the combined application of biochar and limestone^17,52^. Soil pH had a smaller increase with L2 (1.2% lime) compared to the RBL1 (2.5% rice straw biochar + 0.6% limestone) in a pot experiment^52^. In another rice pot experiment, soil pH was maintained similar changes in the two treatments when the amount of rape straw biochar was equal (12 g/kg) and the difference between limestones was doubled (0.3 g/kg, 0.6 g/kg)^17^. The different results from wheat to rice experiments may be due to different soil water conditions and the ratio of biochar and limestone.

There was a significant decrease in wheat grain yield by application of 2% limestone in the low Cd soil compared to CK (Table 3). It was also reported that the wheat grain yields were markedly reduced by high rate of limestone application (at a rate of 2%) compared to the lower rates (at a rate of 0.5% and 1%) Zhou^9^. Evidences from several studies indicated that adding substantial quantities of limestone can impact the biological availability and utilization of ionic (such as Mg, K, and P), resulting in decreasing crop yield^45^. Thus, the high application rate of amendments would induce yield loss of the crop. More studies are needed to better improve the application of amendments for the reduction of negative effects on soil–plant ecosystems^5^.

Biochar can improve soil productivity. The highest increase in total biomass and grain yield of wheat was obtained by rice husk biochar in low Cd soil which was about 25.6% and 34.0% higher compared to the CK (Table 3). Biochar amendments could improve crop yield by acting as a direct nutrient source and by improving soil pH, CEC, surface interactions, and so on^53,54^. In a pot experiment on wheat^55^, soil organic matter content was more effectively increased by application of rice husk biochar than rice straw or rice husk due to the recalcitrance of C-organic in rice husk biochar^56,57^ Rice husk biochar supplies soil nutrients especially some primary macroelements (et. potassium) and increase the fertility of the soil to increase crop yield. Moreover, it is worth mentioning that rice husk biochar is the richest source of Si that through pyrolysis can be increasing of solubility of Si^58^ and promotes plant growth^40^.

Overall, this study found that effects of rice husk biochar on plant growth and Cd accumulation of wheat were better than limestone and rice husk biochar + limestone under the same rate application. As a result, the general effect of 2% rice husk biochar was best and conducive to application in field production.

## Conclusion

The application of the amendments positively influenced the studied wheat and soils. The maximum increase in soil pH was obtained by limestone which was about 35.3% higher compared to the control. Rice husk biochar was most effective on increase of wheat grain yield and decrease of grain Cd content that increased by 25.6% and decreased by 34.0%, respectively. The effect of combined application of amendments was poorer probably due to sub- optimal ratio of the biachar and limestone. Future works are needed to explore the adsorption mechanism of Cd by rice husk biochar and the optimal proportion of combined amendments.

## Data Availability

All methods were performed following the relevant guidelines and regulations. The raw data supporting the conclusions of this article are available from the corresponding author upon reasonable request.
